# Transcription profiles of skin and head kidney from goldfish suffering hemorrhagic septicemia with an emphasis on the TLR signaling pathway

**DOI:** 10.24272/j.issn.2095-8137.2019.028

**Published:** 2019-07-18

**Authors:** Jian-Peng Chen, Wei Pang, Zi-Wei Zhao, Yan-Hui Bi, Xiao-Wu Chen

**Affiliations:** 1 National Demonstration Center for Experimental Fisheries Science Education, Shanghai Ocean University, Shanghai 201306, China; 2 Shanghai Engineering Research Center of Aquaculture, Shanghai 201306, China; 3 Shanghai Collaborative Innovation for Aquatic Animal Genetics and Breeding, Shanghai 201306, China

**Keywords:** *Carassius auratus*, Head kidney, Hemorrhagic septicemia, Illumina sequencing, Skin

## Abstract

Hemorrhagic septicemia is an acute, highly fatal disease that affects goldfish (*Carassius auratus*). To gain a better understanding of related immune genes, the transcriptomes of the skin and head kidney of goldfish suffering hemorrhagic septicemia were sequenced, assembled, and characterized. Based on functional annotation, an extensive and diverse catalog of expressed genes were identified in both the skin and head kidney. As two different organs, pair-wise comparison identified 122/77 unigenes up/down-regulated (two-fold change with *P*<0.05) in the skin and head kidney. Most genes of the immune pathways were expressed and isolated in both skin and head kidney, including interferon (IFN) transcription factors 1–10 and Toll-like receptors (TLRs). Interferon regulatory factor 3 (IRF3), a key IFN transcription factor, was up-regulated at the transcriptional level by polyriboinosinic: polyribocytidylic acid (poly I:C) challenge and regulated the IFN response by increasing the activity of IFN-β and IFN-stimulated response element (ISRE)-containing promoter. This study will benefit the identification and understanding of novel genes that play important roles in the immunological reactions of fish suffering from hemorrhagic septicemia.

The goldfish is not only an important ornamental and aquacultural fish in China, but also one of the most common aquarium fish worldwide (Shin & Choi, 2014). Rapid development of large-scale and highly intensive goldfish farming has increased outbreaks of disease, resulting in serious effects on aquaculture production (Choe et al., 2017). Hemorrhagic septicemia is often caused by stress and overcrowding. Both the innate and acquired immune pathways may be initiated in fish with hemorrhagic septicemia (Montero et al., 2009).

Teleosts possess both innate and adaptive immunity (Vasta et al., 2011). In addition, signal transduction in regard to immunity has been studied in mammals intensively. For example, in the Toll-like receptor signaling pathway, the innate immune system responds to pathogenic microorganism invasion using TLRs via recognition of specific molecular patterns present in microbial components. Therefore, distinct patterns of gene expression induced by the stimulation of different TLRs result not only in the activation of innate immunity but also the development of acquired specific antigen immunity (Akira & Takeda, 2004). Increased expression of immune genes such as IRF 3, IRF 7, IFN-stimulated genes (ISG)15, Type I IFN, and Interferon-induced GTP-binding protein Mx has been observed when viral copy numbers of the hemorrhagic septicemia virus (VHSV) are high, thus revealing their important roles in host defense against VHSV (Avunje et al., 2011). To the best of our knowledge, no related data have been reported regarding gene expression patterns related to hemorrhagic septicemia in goldfish. The identification of genes from infected fish could assist in the discovery of a cure for the disease. As a physical barrier in fish, the skin contains natural antibodies and protects against causative agent penetration (Bos & Kapsenberg, 1993; Hatten et al., 2001). Skin is considered the largest immunologically active organ in fish and differs from that of terrestrial vertebrates. A comprehensive understanding of fish skin is important for the development of new products such as mucosal vaccines aimed at improving the health of cultured fish (Beck & Peatman, 2015). The main cells found in the head kidney are macrophages, which aggregate into structures called melanomacrophage centers, and lymphoid cells, which are found at all developmental stages and exist mostly as B cells (Kobayashi et al., 2006).

In this study, one-year-old water-cultured goldfish were clinically diagnosed with hemorrhagic septicemia based on external reddening and hemorrhaging. Healthy fish, identified by their appearance and activity, were collected and randomly separated into two groups. The control-group was intraperitoneally injected with sterile phosphate buffer solution (PBS) and the treatment-group was injected with poly I:C (0.5 µg/g body weight; Sigma-Aldrich, USA) dissolved in sterile PBS. Organs, including skin, brain, liver, stomach, head kidney, ovary, and testes, were obtained from five individuals in each group 12 h after poly I:C treatment.

Trizol reagent (Invitrogen, USA) was used to extract total RNA as per the manufacturer’s instructions. Genomic DNA of zebrafish was obtained from skeletal muscle tissue samples using the phenol/chloroform method.

An Illumina HiSeq library was constructed following the manufacturer’s instructions. Multiplexed cDNA libraries mixed with a normalized concentration of 10 nmol/L were developed. The HiSeq 2000 platform (Illumina Inc., USA) was then used to sequence the library with the paired-end approach in a single run.

Stringent filtration and *de novo* assemble of the mixed raw sequencing reads from three samples were performed. The contigs assembled from all clean reads with Trinity software were then employed to estimate protein-coding regions through the GetORF module of the EMBOSS package (Itaya et al., 2013). Whether the read count for a given gene could be mapped depended on its sequence length and sequencing depth, with gene expression standardized by reads per kb per million reads (RPKM) (Mortazavi et al., 2008). The expression levels of all genes were compared (via RPKM) using MA plot-based DEGseq with random sampling (Wang et al., 2010). The threshold of the *q* value was determined by the Benjamini-Hochberg method with multiple tests. In the present study, “*q*<10*^-^* ^3^” and “|log2 (RPKM a/RPKM b)|≥2” were used to judge organ-biased genes.

Quantitative polymerase chain reaction (qPCR) was carried out to measure *irf3* gene expression with the iTaq Universal SYBR Green Supermix (Bio-Rad, USA) package, as per our previous study (Wang et al., 2016). The qPCR primers are listed in Supplementary Table S1.

The full-length cDNA of goldish IRF3 was amplified with two pairs of primers, IRF3-ORF and IRF3-FLAG, using nested PCR, followed by cloning and insertion into the pCMV3-FLAG plasmid (Sigma) using double-enzyme restriction. In the same manner, the zebrafish IFN–β promoter was cloned and inserted into the luciferase reporter vector PGL3-Basic with the primer DrIFNβ-promoter. The primers used are listed in Supplementary Table S1. In the IRF3-pCMV3-FLAG plasmid, FLAG tag was merged with the N-terminal of IRF3.

Transfection of human 293T cells with plasmid and Lipofectamine 2000 was performed following the manufacturer’s protocols. The 293T cells were seeded onto 24-well plates and cotransfected with different doses of pCMV3-FLAG-IRF3 together with 100 ng of zebrafish IFN–β promoter luciferase reporter plasmid and 10 ng of pRL-TK (Promega, USA).

After 24 h post-transfection and lysis of 293T cells with Reporter Lysis Buffer (Promega, USA), luciferase activity was evaluated with Dual-Luciferase Assay Reagent (Promega, USA). In addition, the human 293T cells were transfected with different doses of pCMV3-FLAG-IRF3 plasmid (0, 200, 400, and 600 ng) and an equal amount of internal reference pRL-TK plasmid (10 ng). Western blotting was performed as per our previous study (Gu et al., 2016).

Sequencing of the two libraries derived from the two organs generated 32 554 292 and 39 305 131 raw reads, encompassing 14.37 Gb of sequences, which is enough for quantitative analysis of gene expression profiles. After trimming for *de novo* assembly, we obtained 28 646 938 (87.99%) and 34 682 151 (88.24%) clean reads from the skin and head kidney, respectively. All reads were assembled into 30 119 unigenes ranging from 201 bp to 36 481 bp, with an average length of 2 004 bp and N50 length of 2 906 bp (Table 1). The raw sequence data from transcriptome shotgun assembly (TSA) were deposited in GenBank (accession No. GBZM00000000.1).

**Table 1:** Summary statistics of RNA-seq data

	Total	
	Skin	Head kidney
**Obtained from sequencing**		
Reads	32 5542 92	39 305 131
Nucleotides (bp)	6 510 858 400	7 861 026 200
**After trimming for de novo assembly**		
Reads	28 646 938	34 682 151
Nucleotides (bp)	5 529 141 837	6 692 024 669
**De novo assembly**		
**Contigs**		
Total length (bp)	157 725 712	
Sequence number	488 213	
Longest sequence length (bp)	36 468	
Average length (bp)	323	
N50	467	
**Transcripts**		
Total length (bp)	341 412 899	
Sequence number	256 284	
Longest sequence length (bp)	36 481	
Average length (bp)	1 332	
N50	2 603	
**Unigene**		
Total length (bp)	60 368 848	
Sequence number	30 119	
Longest sequence length (bp)	36 481	
Average length (bp)	2 004	
N50	2 906	

Results showed that 751 and 1 249 unigenes were unique to the skin and head kidney, respectively (Figure 1A). In addition, pair-wise comparisons revealed that 112/77 unigenes were up/down-regulated by at least a two-fold change between the skin and head kidney (Figure 1B). To characterize these differentially expressed genes (DEGs) between the skin and head kidney, we conducted GO annotation and KEGG pathway analyses. The GO annotation results showed that the DEGs could be assigned into 74, 17, and 15 categories of biological process, cellular component, and molecular function, and subsequently separated into 104 subcategories. Subcategories of “cytoskeleton organization”, “transport”, and “cellular component” were the main gene enrichment categories. The differentially expressed unigenes belonged to 51 KEGG subcategories (Figure 1C).

**Figure 1:**
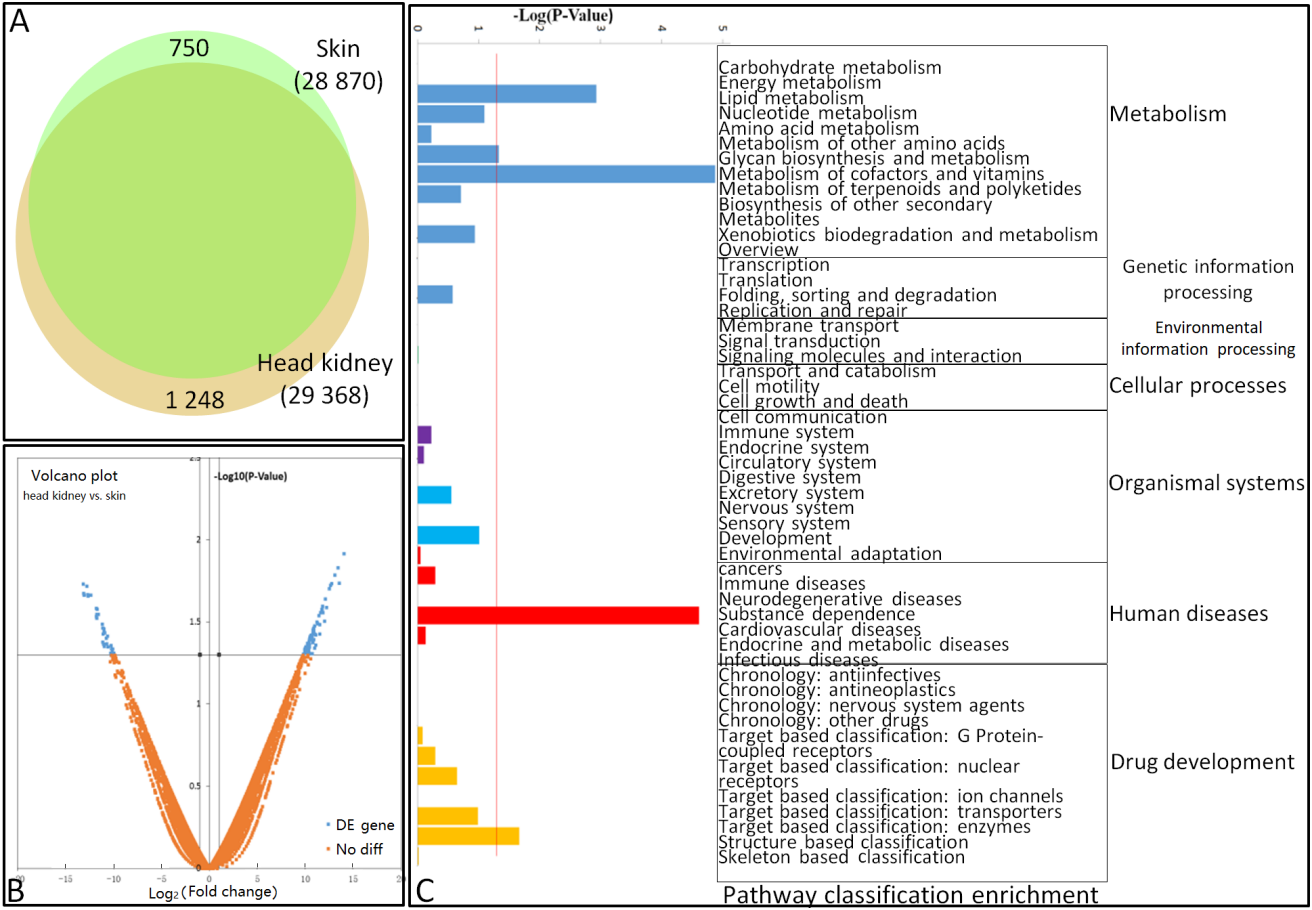
Differential gene expression profiles of goldfish skin and head kidney A: Venn diagram showing number of total genes expressed in skin and head kidney. B: Volcano plot of differentially expressed genes. Scatter-plot of –log_10_^(P-Value)^ against log_2_^(Fold Change)^ expression in head kidney/skin. In total, 77 genes up-regulated on left (blue) and 112 genes down-regulated on right (blue) (FDR<0.001). C: KEGG pathway classification of DEGs.

We identified 1 475 proteins associated with immune function. Furthermore, 221 proteins, accounting for 15% of total selected proteins, were identified in the transcriptome as belonging to the chemokine signaling pathway. As the result of the inflammatory response, chemokines are released from various cells after bacterial infection. Here, 57 genes from the Toll-like receptor signaling pathway were expressed in both skin and kidney (Supplementary Figure S1). In addition, as IFN transcription regulators, the IRF family (IRF1~10) and TLR1 were selected for reverse transcription PCR (RT-PCR), as those genes are expressed in different organs, including the skin and head kidney (Figure 2A). Based on RNA-seq and RT-PCR analyses, the goldfish skin and head kidney were both found to express important immune genes. Determination of differences in mRNA expression level in goldfish IRF3 was carried out using RT-PCR for the two organs in the diseased, poly I:C-treated, and control fish. After 12 h stimulation with poly I:C, mRNA expression was increased significantly in the skin and head kidney; however, the increase was not significantly different between the poly I: C-treated and diseased fish (Figure 2B).

**Figure 2:**
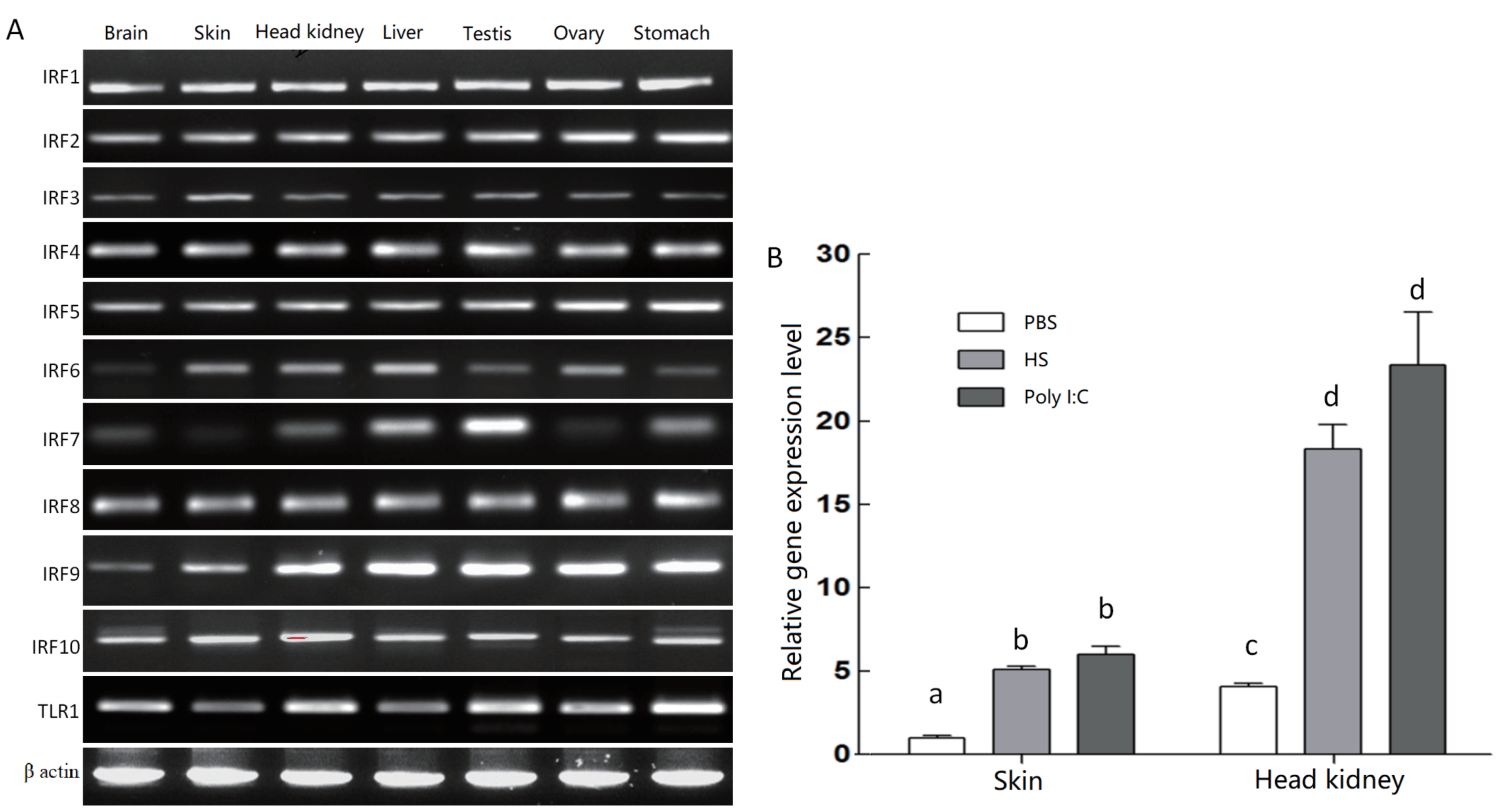
mRNA expression analysis of several genes from TLR signaling pathway A: Reverse transcription-polymerase chain reaction analysis of 11 genes from interferon synthesis-related pathways in different organs, *β*-actin was constitutively expressed and used as a control to evaluate integrity of RNA and efficiency of RT-PCR. B: qPCR analysis of IRF3 expression in skin and head kidney from different groups. *β*-actin was used as a control to evaluate amount and quality of RNA. Densitometry values are means±*SD*. Different lowercase letters indicate significant differences (*P*<0.05). HS: Hemorrhagic septicemia-infected fish group.

The typical zebrafish interferon–β promoter dual luciferase reporter assay was carried out to evaluate the relationships among goldfish IRF3 (Figure 3A). As shown in Figure 3A, transfection of Irf3 expression plasmid was demonstrated in a dose-dependent manner (Figure 3B). Interferon–β promoter activity was significantly activated by the over-expression of goldfish IRF3 in the 293T cells compared with the empty vector (*P*<0.05) (Figure 3C). This indicated that goldfish IRF3 likely participates in IFN signaling in innate immunity.

**Figure 3:**
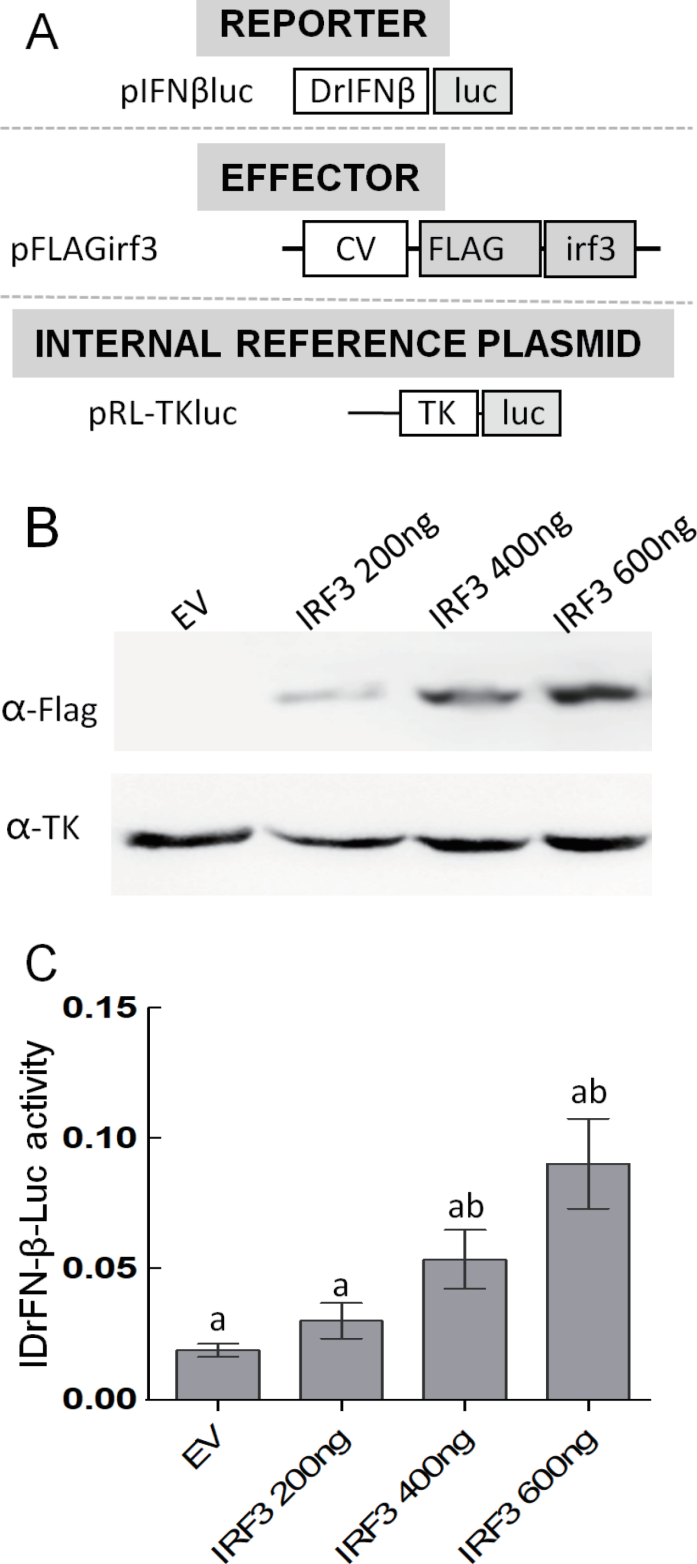
Luciferase reporter assay A: Reporter and effector plasmids. CV: Human cytomegalovirus immediate early enhancer/promoter; DrINFβ: Zebrafish interferon β promoter (first letter of initiation codon ATG is defined as +1, nucleotides from -2 000 to +1 were cloned into the luciferase reporter plasmid); TK: Human herpes simplex virus thymidine kinase promoter; FLAG: FLAG tag; irf3: medaka irf3 cDNA; luc: firefly luciferase. B: Western blotting analysis of Irf3 expression in cells at different plasmid concentrations. C: Goldfish IRF3 activation of IFNβluc. pFLAG empty plasmid (EV; negative control) and luciferase reporter plasmids pIFNβluc or pISREluc plus pRL-TK (internal control reporter) were co-transfected into 293T cells. At 24 h post-transfection, cells were lysed for luciferase assay. Different lowercase letters indicate significant difference at *P*<0.05.

Skin is the first immune barrier and effectively eliminates risk from outside pathogenic organisms. As an active immunological microenvironment, it is quite different from other barrier tissues between the body and exterior environment (Bos & Kapsenberg, 1993).

The skin mucus consists of several defensive systems, including antibody (Palaksha et al., 2008) and innate and acquired immune systems (Alvarez-Pellitero, 2008). Human skin transcriptome reveals many immune genes associated with epidermal wound healing, such as up-regulated IRF1, IRF7, IRF8, and interferon receptors (Nuutila et al., 2012). Next-generation sequencing identified over 600 000 reads assembled into 34 696 transcripts from the skin of Atlantic salmon (*Salmo salar*), representing a wide variety of genes, including immune-related ones (e.g., cytokines, chemokines, lectins, and interleukins) (Micallef et al., 2012).

The head kidney forms the front part of the fish kidney and undertakes immune functions much like mammalian bone marrow, e.g., hematopoiesis (Press & Evensen, 1999). The head kidney in fish is a basic organ forming blood elements, thus it is potentially useful for identifying new immune-related genes (Gerdol et al., 2015). Moreover, it is a fundamental organ of the fish immune system, and a source of diverse cell effector types and of hematopoietic stem cells in teleosts (Kobayashi et al., 2006). It has been reported that many unigenes from the head kidney in grass carp are involved in the Toll-like, RIG-I-like, and RIG-I-like receptor signaling pathways (Chen et al., 2012). Furthermore, gene expression of pro-inflammatory cytokines, including TNF–α, TNF–β, IL-6, IL-17A/F3, and IFN-γ, and other cytokines, including IL-4/13A, IL-4/13B, and Type I-IFN, is increased in particulate silica-stimulated head kidney cells in Japanese pufferfish (*Takifugu rubripes*) (Morimoto et al., 2016).

Between the two organs, we identified 750 (2.53%) and 1 248 (4.34%) genes showing organ-specific expression, with 112 and 77 genes highly and lowly expressed in the skin and head kidney, respectively. The main reason for this variance may be their different cell compositions, thus indicating possibly different reaction mechanisms to the same pathogen from different organs.

In this study, a set of genes associated with the immune response were expressed in both organs. Similar findings have been presented in previous human studies (Gerdol et al., 2015; Nuutila et al., 2012). As an important innate immune pathway, the Toll-like receptor signaling pathway was selected here for further study. All IRF family members and TLRs were expressed extensively in the two organs. To some degree, these results indicate that the goldfish skin and head kidney react to external stimuli by expressing immune genes and other cytokines to participate in the immune response.

In mammals, an IFN response is triggered by the activation of IRF3, a downstream adaptor of the TLR3 signaling pathway, following stimulation with poly I:C (Taniguchi et al., 2001). In this study, similar to IRF3 in other fish species (Gu et al., 2016; Holland et al.; Sun et al., 2010), the significant induction of IRF3 in goldfish by poly I:C stimulation indicated that IRF3 might play an important role in virus infection. It has been reported that mammalian IRF3 is a key transcriptional factor in the mediation of the type I IFN-dependent immune response against DNA or RNA-virus infection (Honda & Taniguchi, 2006). Mammalian IRF3 activates the mRNA transcription of type I IFN genes (IFN–α and –β) and ISG by binding to the ISRE on their promoters (Honda & Taniguchi, 2006; Paun & Pitha, 2007). Recently, it has been confirmed that IRF3 in fish plays a potential role in the IFN response as IFN-β and ISRE-containing activities can be induced by the over-expression of IRF3 (Holland et al., 2008; Sun et al., 2010). The characterization of the IRF3 function domain and positive relationship between IFN-β and IRF3 revealed by the luciferase reporter assay in this study suggests that goldfish IRF3 functions as a key regulator in the IFN response signaling pathway.
